# The effect of cognitive behavioral therapy on future thinking in patients with major depressive disorder: A randomized controlled trial

**DOI:** 10.3389/fpsyt.2023.997154

**Published:** 2023-01-25

**Authors:** Mizuki Amano, Nariko Katayama, Satoshi Umeda, Yuri Terasawa, Hajime Tabuchi, Toshiaki Kikuchi, Takayuki Abe, Masaru Mimura, Atsuo Nakagawa

**Affiliations:** ^1^Department of Neuropsychiatry, Keio University School of Medicine, Tokyo, Japan; ^2^Department of Psychology, Faculty of Letters, Keio University, Tokyo, Japan; ^3^Graduate School of Data Science, Yokohama City University, Yokohama, Japan; ^4^Department of Neuropsychiatry, St. Marianna University School of Medicine, Kawasaki, Japan

**Keywords:** future thinking, cognitive behavioral therapy, major depressive disorder, future thinking task, response time

## Abstract

**Background:**

Pessimistic thinking about the future is one of the cardinal symptoms of major depression. Few studies have assessed changes in pessimistic thinking after undergoing cognitive behavioral therapy (CBT). A randomized clinical trial (RCT) was conducted with patients diagnosed with major depressive disorder (MDD) to determine whether receiving a course of CBT affects pessimistic future thinking using a future thinking task.

**Methods:**

Thirty-one patients with MDD were randomly assigned to either CBT (*n* = 16) or a talking control (TC) (*n* = 15) for a 16-week intervention. The main outcomes were the change in response time (RT) and the ratio of the responses for positive valence, measured by the future thinking task. Secondary outcomes included the GRID-Hamilton Depression Rating Scale, the Beck Depression Inventory-Second Edition (BDI-II), the Dysfunctional Attitude Scale (DAS), and the word fluency test (WFT).

**Results:**

Regarding the main outcomes, the CBT group showed reduced RT for the positive valence (within-group Cohen’s *d* = 0.7, *p* = 0.012) and negative valence (within-group Cohen’s *d* = 0.6, *p* = 0.03) in the distant future condition. The ratio of positive valence responses in both groups for all temporal conditions except for the distant past condition increased within group (distant future: CBT: Cohen’s *d* = 0.5, *p* = 0.04; TC: Cohen’s *d* = 0.8, *p* = 0.008; near future: CBT: Cohen’s *d* = 1.0, *p* < 0.001; TC: Cohen’s *d* = 1.1, *p* = 0.001; near past: CBT: Cohen’s *d* = 0.8, *p* = 0.005; TC: Cohen’s *d* = 1.0, *p* = 0.002). As for secondary outcomes, the CBT group showed greater improvement than the TC group regarding the need for social approval as measured by the DAS (*p* = 0.012).

**Conclusion:**

Patients with MDD who received CBT showed a reduced RT for the positive and negative valence in the distant future condition. RT in the future thinking task for depressed patients may be a potential objective measure for the CBT treatment process. Because the present RCT is positioned as a pilot RCT, a confirmatory trial with a larger number of patients is warranted to elucidate the CBT treatment process that influences future thinking.

**Clinical trial registration:**

https://center6.umin.ac.jp/cgi-open-bin/icdr_e/ctr_view.cgi?recptno=R000021028, identifier UMIN000018155.

## 1. Introduction

Patients suffering from major depressive disorder (MDD) commonly demonstrate pessimistic and negative views toward the future. Pessimistic thinking about the future is one of the cardinal symptoms of MDD and an essential domain of the psychopathology of depression (1, 2). Beck’s cognitive theory of depression is a widely known stress-vulnerability model explaining negative-biased views of the future as well as oneself and the world (*the negative triad*) that posits a major role in the onset, maintenance, and recurrence of depression. Beck asserted that this negative triad is based on the automatically activated dysfunctional self-schema, a fundamental cognitive structure that processes information about oneself through screening, differentiating, and coding (2). Patients with depression generally show a limited adaptive cognitive style, which is characterized by abstract and over-generalized processing linked to a poor evaluation of possible futures, which develops hopelessness. Hopelessness has been associated with suicidal behavior (3), for which cognitive behavioral therapy (CBT) has potential effect (4). Further, depressed patients have a diminished ability to imagine a positive future (5).

Diminished ability to imagine a positive future is a specific feature of future thinking (6). Depressed individuals anticipate fewer positive events than healthy individuals (7). Bjärehed et al. (8) reported a decrease in positive expectations among depressed individuals but no increase in negative expectations based on the future thinking task (FTT), a measure of future thinking based on the verbal fluency paradigm (9). Kosnes et al. (10) indicated a decrease in positive expectations for the future based on the explicit FTT, and the implicit FTT was more pronounced as measured by response time. Our previous study reported that the FTT response time for the positive valence responses in the distant future condition was prolonged in patients with MDD (11). Thus, future thinking in depression may be characterized as a diminished ability of positive anticipation. Perhaps patients with MDD experience difficulty in imagining their positive distant future, which may lead to engaging more frequently with pessimistic thinking. Although future thinking has been extensively studied (12) and several studies have reported that verbal fluency tasks, verbal fluency paradigms and response times using verbal fluency paradigms are associated with hopelessness and suicidal behavior (5, 10, 13). Few studies have examined changes after CBT) for patients with depression. One study found that depressed patients engaged in more positive future thinking after administration of CBT (14), and this was not observed among those who received routine medication treatment (5). In another study, FTT index scores for negative events were reduced after CBT, and change scores for the FTT negative events correlated with depression symptoms (15). Thus, alleviating pessimistic future thinking through improving positive anticipation may help depressed patients imagine their positive distant future and specifically reduce the response time of the distant future condition on the FTT.

Few studies have evaluated the change in future thinking among patients with MDD who received a course of CBT. The present study was conducted at a tertiary care hospital where MDD patients have been visiting and seeking care because they had not responded adequately to initial antidepressant treatment. Therefore, a randomized clinical trial (RCT) was conducted with patients diagnosed with moderate to severe MDD to determine whether receiving a course of CBT affects the response time and percentage of positive recall responses to four temporal conditions in the FTT differently than talking control (TC). We also explored the relationship between the changes in future thinking and the dysfunctional schema.

## 2. Materials and methods

### 2.1. Study design

The present study was a 16-week RCT of two parallel groups with a 12-month follow-up. The full RCT protocol has been published elsewhere (16). This study was registered with the UMIN Clinical Trials Registry (identifier: UMIN000018155) and approved by the Ethical Review Committee of Keio University School of Medicine (reference No. 20150070). The neural change and functional connectivity outcomes have been reported elsewhere (17); this article focuses on the behavioral outcomes.

### 2.2. Patients

From July 2015 to October 2019, patients were recruited from a university teaching hospital located in Tokyo, Japan. Those who agreed to participate were asked to provide written informed consent prior to initiating any study procedure.

Eligible participants were outpatients aged 20–69 years, had a diagnosis of MDD as defined by the Diagnostic and Statistical Manual of Mental Disorders, Fourth Edition (DSM-IV) (18), either single or recurrent episodes, and without psychotic features. They were assessed by trained psychiatrists using the DSM-IV Structured Clinical Interview (19), with a total score of 16 or higher on the 17-item GRID-Hamilton Depression Rating Scale (GRID-HAMD17) (20).

### 2.3. Interventions

Patients received a course of either CBT or TC sessions with therapists and routine psychiatric clinical management. Patients were randomized 1:1 to CBT or TC after the baseline assessment. Allocation was concealed with the use of a computer-generated random allocation system.

#### 2.3.1. Cognitive behavioral therapy (CBT)

Patients allocated to the CBT group were offered 16 individual 50-min sessions, scheduled weekly, with up to four additional sessions if deemed clinically appropriate by the therapist. Therapists followed the procedures outlined in the CBT Manual for Depression (available at the Japanese Ministry of Health, Labour and Welfare website^1^). This manual is based on Beck’s original treatment manual (2), with some adaptions designed to address the cultural characteristics of Japanese patients (21). Six therapists, a psychiatrist (*n* = 1), a psychiatric nurse (*n* = 1), or masters- or doctoral-level clinical psychologists (*n* = 4) provided CBT. They had practiced CBT for a mean of 5.5 (SD = 4.1) years and had used CBT to treat a mean of 15.7 (SD = 13.9) patients prior to this study. All received CBT training, including a 2-day intensive workshop plus weekly 1-h on-site group supervision sessions with a skilled CBT supervisor (AN), with thorough review of the cases and detailed feedback to maintain adherence to CBT protocols and competence throughout the study period.

#### 2.3.2. Talking control (TC)

Participants allocated to the TC group were offered a talking control based on the manual of Serfaty et al. (22). TC is an unstructured supportive therapy to encourage patients to talk about their daily experiences and offer empathy without trying to solve problems or teaching new skills. Clearly defined criteria for the TC group were used to prevent CBT from being delivered. TC was also delivered by the same six therapists who provided the CBT in line with previous studies (23). All therapists had practiced psychotherapy including supportive therapy for a mean of 4.5 (SD = 1.7) years and had sufficient clinical experience and training in the elements of supportive therapy. The therapists received TC training, including a 1-day intensive workshop, and practiced delivering the TC in 3-h role plays with the supervisor so that difficult questions could be addressed. One hour of group supervision was then conducted every week, and audiotapes of therapy sessions were evaluated using a fidelity checklist to maintain adherence to TC protocols and competence during the study.

### 2.4. Outcome Measures

Our main outcomes were the change in response time (RT) and the ratio of the number of responses for positive valence using the FTT at 16 weeks. Valence indicated the emotional value that was associated with events or situations (24). Positive valence refers to responses in a positive context, and negative valence refers to responses in a negative context. Secondary outcomes were the change in scores for depression symptoms as measured by the GRID-HAMD17 and the Beck Depression Inventory-Second Edition (BDI-II), dysfunctional schemas as measured by the Dysfunctional Attitude Scale (DAS), and neurocognitive function as measured by the word fluency test (WFT).

#### 2.4.1. Main outcomes

**FIGURE 1:**
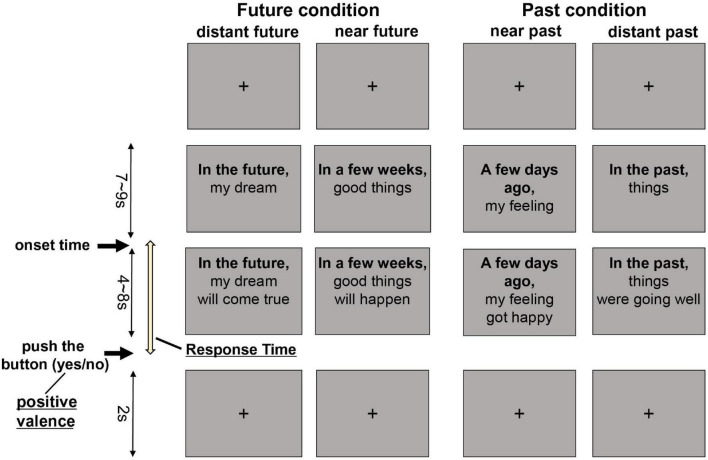
Future thinking task. Example trials for four different conditions are shown. Participants were asked to imagine the future or recall the past when presented with a temporal word on a slide. Once the sentence was complete, participants pushed a button to indicate a response of yes or no.

A schema of an example trial is shown in Figure 1. Based on the Future Thinking Implicit Relations Assessment Procedure (10), we used the FTT contextualized and adapted to the Japanese context (11, 25). The FTT consisted of four temporal conditions, which were categorized into two groups relating to temporal directions (past/future) and two groups relating to distance (near/distant). Each trial began with a description of the temporal words (e.g., “in the future”) and their primary context (e.g., “your dream”). Participants were asked to imagine events in the distant or near future or to remember events in the distant or near past. When presenting a complete sentence (e.g., “in the future, your dream will come true”), patients were required to press a button to respond “yes” or “no” after judging whether the content was congruent with what they were thinking. The FTT included 64 trials, which took the time ranging from 21.6 to 25.0 min. Stimuli were presented using SuperLab software (version 5.0; Cedrus Corp., San Pedro, CA, USA), and the respondent’s RT and answer were collected.

All patient responses indicated a positive or negative valence to each temporal condition. The change in the ratio of the number of responses for positive valence in each temporal condition was calculated to assess the severity of negative future thinking. RT was measured from when the whole sentence was shown to when the patient pushed the button to respond. The ratio of the number of responses for positive valence in each temporal distance condition was calculated to assess differences between the groups. All stimuli were presented in black text on a light white background and projected on a screen viewed by participants on a mirror in the MRI.

#### 2.4.2. Secondary outcomes

Outcomes were collected at baseline and 16 weeks. Alleviation of depressive symptoms was measured by the change in the GRID-HAMD17 score and patient-reported measures of depressive symptoms using the BDI-II (26). Improvement of dysfunctional schema was measured by a change in the DAS (27) total score and three subscale scores (Achievement, Self-Control, and Need for Social Approval) (28). Assessment of the levels of word fluency was carried out by the WFT (29). All assessors received extensive GRID-HAMD17 training, exhibiting excellent interrater reliability with an intraclass correlation coefficient of 0.94–0.98.

All GRID-HAMD17 assessors were masked to the allocated treatment. Considering the percentage of agreement and κ coefficients between the actual allocation and the allocation guessed through asking the masked assessors at the post-treatment visit (at 16 weeks) were 54.0% and 0.093 (95% confidence interval [CI]: −0.36 to 0.36), respectively, the masking was successful.

### 2.5. Sample size estimation

Because this trial was a pilot RCT with a behavioral experiment conducted under MRI, the sample size was determined primarily based on feasibility considerations. Therefore, we set a target number of 19 participants per group (38 total). The details are reported elsewhere (17).

### 2.6. Statistical analysis

Demographic factors and patients’ baseline characteristics were summarized by each treatment group. Data were presented with mean ± standard deviation. Analyses were performed in the per-protocol sample, including participants completing the future thinking task. Treatment effects on the FTT were evaluated with the *t*-test for mean change from baseline between groups and paired *t*-test (null hypothesis: mean change = 0) for the pre and post values within groups. Clinical and neurocognitive variables were evaluated with the *t*-test for mean change from baseline in outcomes. Pearson’s correlation coefficients between change in a variable in the FTT and change in DAS were calculated. For all analyses, the significance level was set at 0.05 (two-sided). All statistical analyses were performed using SPSS (version 27.0).

## 3. Results

### 3.1. Patient characteristics

**FIGURE 2:**
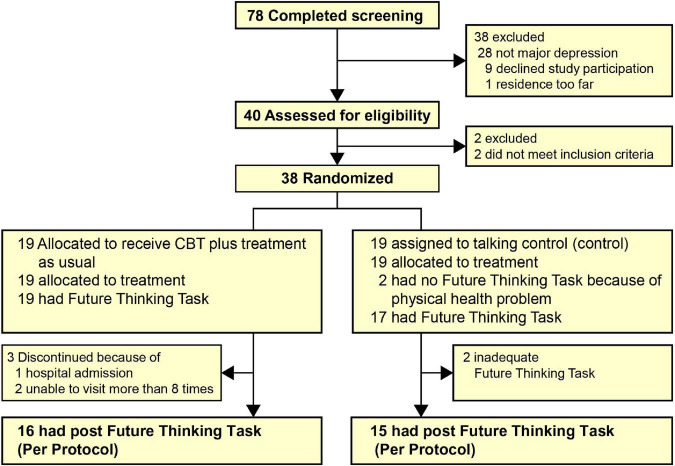
CONSORT diagram of the participant flow of the study. CBT, cognitive behavioral therapy.

Figure 2 shows the flow of the patients from screening to post-treatment (16 weeks). Thirty-eight patients were randomized to receive CBT (*n* = 19) or TC (*n* = 19). Of those randomized, 17 in the CBT group and 18 in the TC group completed a 16-week intervention. Of the 35 post-treatment assessment completers, 31 (CBT = 16, TC = 15) completed the future thinking task. Table 1 shows the baseline demographic and clinical characteristics of the treatment groups. Typically, patients were in their 30–40s, had two previous depression episodes, had been in the current episode for nearly 5 years, and were severe to moderately depressed. None of the patients experienced serious adverse events during the intervention period.

**TABLE 1:** Participant characteristics at baseline.

Characteristics	CBT (*N* = 19)		TC (*N* = 19)	
Demographic characteristics				
	Mean	SD	Mean	SD
Age (years)	38.0	7.4	36.9	10.1
Education (years)	16.3	2.1	15.5	1.4
	***N***	**%**	***N***	**%**
Male gender	9	47.4	9	47.4
Unemployed	0	0	2	10.5
Absences from work	13	68.4	11	57.9
**Marital status**				
Married	10	52.6	9	47.4
Separated, divorced, widowed	2	10.5	0	0
Single	7	36.8	10	52.6
Cohabiting (yes)	15	78.9	18	94.7
Smoking habit (yes)	5	26.3	3	15.8
Alcohol habit (yes)	10	52.6	11	57.9
**Clinical characteristics**				
Previous hospitalization	1	5.3	1	5.3
Previous suicide attempts	1	5.3	3	15
Self-reported childhood abuse	2	10	0	0
Self-reported experience of childhood bullying	3	15	7	36
Family history of psychiatric disorders (first-degree)	10	52	5	26
**Specifiers of index episode (DSM-IV)**				
Chronic (≥2 years from index episode)	10	52.6	10	52.6
Melancholic features	3	15	2	10
Atypical features	0	0	1	5.3
**Comorbid DSM-IV Axis I diagnosis**				
Panic disorder (with agoraphobia)	1	5.3	2	10.5
Social anxiety disorder	1	5.3	0	0
Obsessive-compulsive disorder	0	0	0	0
Generalized anxiety disorder	1	5.3	0	0
Dysthymic disorder	0	0	1	5.3
	**Mean**	**SD**	**Mean**	**SD**
Total number of depression episodes	2.0	0.94	2.2	1.6
Duration of index depression episode (months)	67.5	74.5	63.9	86.7
DDD of antidepressant medications prescribed at baseline	1.1	0.49	1.1	0.63
**Depression severity**				
GRID-HAMD_17_	20.0	4.0	21.3	5.6
BDI-II	29.3	8.0	28.9	9.9
**Dysfunctional cognition**				
DAS	117.2	23.0	104.7	18.1
**Neurocognitive function**				
**Word fluency**				
Initial words	25.5	5.6	26.8	8.0
Category	42.2	7.7	43.2	7.7

CBT, cognitive behavioral therapy; TC, talking control; MDD, major depressive disorder; DDD, defined daily dose; GRID-HAMD_17_, 17-item GRID-Hamilton Depression Rating Scale; BDI-II, Beck Depression Inventory-II; DAS, Dysfunctional Attitude Scale.

### 3.2. Main outcomes

**FIGURE 3:**
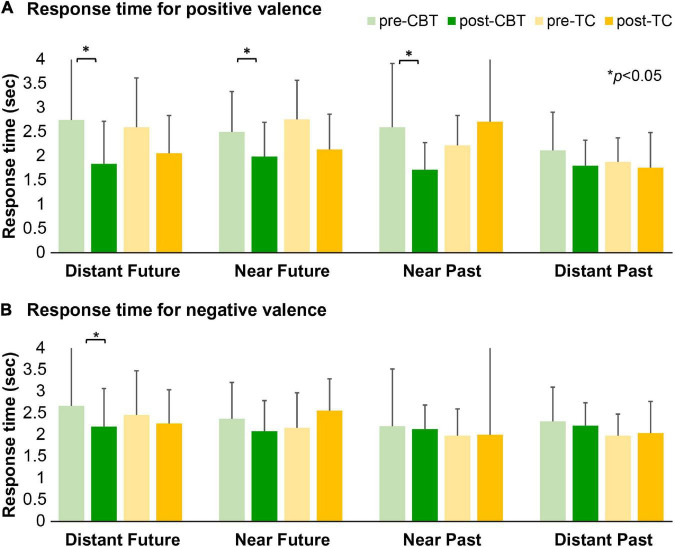
The response time for the future thinking task. Mean RTs for positive **(A)** and negative valence **(B)** across the four different conditions for the CBT and TC groups before and after treatment. CBT, cognitive behavioral therapy; TC, talking control; RT, response time. **(A)** Greater reductions in RTs for both positive valence in the distant future condition (*p* = 0.012), near future condition (*p* = 0.017), and near past condition (*p* = 0.02) in the CBT group. **(B)** A significant reduction in RT for negative valence in the distant future condition in the CBT group (*p* = 0.03).

The results of the RT assessed using the FTT are presented in Figures 3A, B and Supplementary Table 1. Between groups, no significant change from baseline for the RT both of positive and negative valences was observed for any of the temporal conditions. Within-group effects were large in the RTs for both positive (Cohen’s *d* = 0.7, *p* = 0.012) and negative valence (Cohen’s *d* = 0.6, *p* = 0.03) for the distant future condition in the CBT group. There was a reduction of RTs for positive valence for the near future condition (Cohen’s *d* = 0.7, *p* = 0.017) and near past condition (Cohen’s *d* = 0.7, *p* = 0.02) in the CBT group.

**FIGURE 4:**
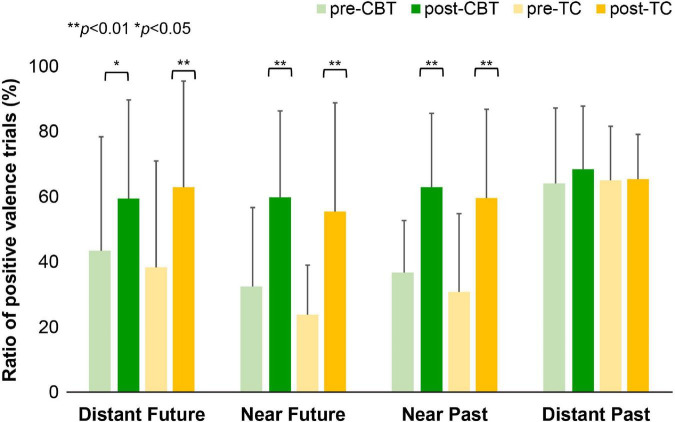
The ratio of positive valence trial in the future thinking task. Mean ratio of positive valence across the four different conditions for the CBT group and TC group before and after treatment. A paired *t*-test shows that the ratio of positive valence responses increased after treatment in both groups in the distant future condition (CBT: *p* = 0.04; TC: *p* = 0.008), near future condition (CBT: *p* = 0.001; TC: *p* = 0.001), and near past condition (CBT: *p* = 0.005; TC: p = 0.002). CBT, cognitive behavioral therapy; TC, talking control.

The mean ratios of the positive valence responses for each temporal distance are shown in Figure 4 and Supplementary Table 2. Between groups, no significant change from baseline for the ratio of positive valence responses was observed for any of the temporal conditions. Within-group effects were increased in terms of the ratio of positive valence responses for all temporal conditions except for the distant past condition in both groups [distant future condition: CBT: Cohen’s *d* = 0.5, *p* = 0.04; TC: Cohen’s *d* = 0.8, *p* = 0.008; near future condition: CBT: Cohen’s *d* = 1.0, *p* < 0.001; TC: Cohen’s *d* = 1.1, *p* = 0.001, and near past condition (CBT: Cohen’s *d* = 0.8, *p* = 0.005; TC: Cohen’s *d* = 1.0, *p* = 0.002)].

### 3.3. Secondary outcomes

The results of the clinical assessments are presented in Table 2. Between groups, the patients who received CBT showed greater reductions from baseline for the need for social approval as measured by the DAS (*p* = 0.012) compared to those who received TC. Within-group effects were lower in terms of the means of depression severity (GRID-HAMD17: within-group CBT: *p* < 0.001; TC: *p* < 0.001, BDI-II: within-group CBT: *p* < 0.001; TC: *p* < 0.001) and dysfunctional schema (DAS total score: within-group CBT: *p* < 0.001; TC: *p* = 0.001) after treatment in both groups. Within-group effect was increased in terms of the levels of word fluency in the TC group (WFT initial words: within-group TC: *p* = 0.004).

**TABLE 2:** Secondary outcomes for patients receiving cognitive behavioral therapy and talking control.

	Cognitive behavioral therapy (*n* = 16)								Talking control (*n* = 15)									
	Pre		Post		Change (post-pre)		Within groups^†^		Pre		Post		Change (post-pre)		Within groups^†^		Between groups^‡^	
Measures	Mean	SD	Mean	SD	Mean	SD	*t*	*p*	Mean	SD	Mean	SD	Mean	SD	*t*	*p*	*t*	*p*
**Depression severity**																		
GRID-HAMD17	20.6	4.38	11.4	6.7	−9.2	5.2	7.0	**<0.001**	22.1	5.5	11.2	7.5	−10.9	2.2	5.0	**<0.001**	0.7	0.5
BDI-II SCORE	30.4	7.34	18.9	12.3	−11.4	9.8	4.7	**<0.001**	32.3	9.7	18.5	12.0	−13.9	2.8	5.0	**<0.001**	0.7	0.5
**Dysfunctional schema**																		
DAS Total Score	119.5	20.2	102.4	18.9	−17.1	15.8	4.3	**<0.001**	104.3	17.5	93.9	17.7	−10.5	10.3	3.9	**0.001**	−1.4	0.9
DAS Achievement	51.3	11.5	44.1	13.2	−7.1	10.9	2.6	**0.019**	44.1	10.3	40.6	9.2	−3.5	7.8	1.7	0.1	−1.1	0.3
DAS Self-control	28.2	5.1	25.3	4.8	−2.9	3.9	3.0	**0.01**	24.4	6.5	21.6	6.1	−2.8	5.0	2.2	0.05	−0.04	0.9
DAS Need for social approval	28.8	4.5	19.8	3.2	−8.0	3.6	8.8	**<0.001**	23.2	5.0	20.1	3.3	−3.1	6.3	1.9	0.08	−2.7	**0.012**
**Neurocognitive test**																		
WF-initial words	25.0	5.5	26.6	7.0	1.6	5.5	−1.1	0.3	28.1	8.4	34.1	11.6	5.9	6.6	−3.5	**0.004**	−2.0	0.05
WF-category	41.6	8.1	42.1	9.3	0.4	9.9	−0.2	0.9	44.9	8.3	48.3	11.1	3.3	9.2	−1.4	0.2	−0.8	0.4

Bold items are significant at *p* < 0.05. ^†^Paired *t*-test. ^‡^Independent *t*-test; SD, standard deviation; GRID-HAMD_17_, 17-item GRID-Hamilton Depression Rating Scale; BDI-II, Beck Depression Inventory-II; DAS, Dysfunctional Attitude Scale; WF, word fluency test.

### 3.4. Exploratory analysis of correlations between RT for the positive valence of the future and the DAS

We explored whether the changes in RTs for positive valence in the future condition are associated with the changes in scores on the DAS in the CBT and TC groups. In only the CBT group, there was a significant correlation between reduction in RTs in the distant future condition and the DAS Need for Social Approval subscale scores (*r* = −0.6, *p* = 0.01) but not in the TC group (*r* = −0.05, *p* = 0.8). In addition, in only the CBT group, there was a significant correlation between reduction in RTs in the near future condition and the DAS Need for Social Approval subscale scores (*r* = −0.7, *p* = 0.003), but not in the TC group (*r* = −0.05, *p* = 0.8) (Table 3).

**TABLE 3:** Spearman’s correlation coefficient between RT for the positive valence of the future and DAS total and subscale score.

	CBT		TC	
RT-distant future	*r*	*p*	*r*	*p*
DAS Total score	0.06	0.8	0.09	0.8
DAS Achievement	0.1	0.7	0.2	0.6
DAS Self-control	−0.04	0.9	0.3	0.3
DAS Need for Social Approval	−0.6	0.01*	−0.05	0.8
**RT-near future**				
DAS Total score	0.02	0.9	0.2	0.8
DAS Achievement	0.1	0.7	0.3	0.7
DAS Self-control	0.05	0.9	0.04	0.3
DAS Need for Social Approval	−0.7	0.003*	−0.05	0.8

*Statistically significant after Bonferroni correction. RT-distant future, Change in response time for the positive valence of the distant future; RT-near future, Change in response time for the positive valence of the near future; DAS, Change in dysfunctional attitude scale; CBT, cognitive behavioral therapy; TC, talking control.

## 4. Discussion

Patients with MDD who received a course of CBT showed a reduction in RT in the distant future condition, although no significant difference was observed in terms of the change from baseline for RT or the ratio of the number of responses for positive valence between groups. In addition, MDD patients who received CBT showed a decrease in RT in the near future condition for positive valence. Moreover, the ratio of the number of responses for positive valence in the near and distant future condition increased after the intervention in both the CBT and TC groups. In terms of clinical and neurocognitive change, the dysfunctional schema related with need for approval as measured by the DAS Need for Social Approval subscale was found to improve to a greater degree in the CBT group than in the TC group. Furthermore, among the participants in the CBT group, the improvement in terms of dysfunctional schemas related to the need for approval correlated with a reduction in RT for positive valence in the distant and near future condition.

In our samples, RTs decreased in the CBT group for both the positive and negative valence in the distant future condition. This finding is compatible with our previous study that showed that maladaptive activation in the frontopolar cortex (BA10) was associated with pessimistic future thinking during distant future thinking (11) and decreased after a course of CBT (17). Prior research has shown that depressed patients generally experience a form of thinking through abstract and overly general processing (30), that events imagined to occur in the distant future are experienced ambiguously (31), and that such patients are less likely to make concrete assessments about the future (32). In our study, when patients who completed CBT were asked to qualitatively describe what they had learned about CBT, they commented that they were satisfied with the CBT they had experienced and that it helped them think more concretely about the future (33). CBT has a future-oriented treatment structure, such as training clients in goal setting and planning strategies and using behavioral activation to enable patients to schedule enjoyable and fulfilling future experiences (34). Therefore, these findings may suggest that depressed patients were able to smoothly and concretely evaluate the distant future. A further future possibility is that CBT with a future-oriented treatment structure may not only benefit depression but also those suffering from another psychological construct called demoralization, linked to negative future cognitions and consequent suicide (35, 36).

In the near future condition, MDD patients who received CBT showed a reduction in RT for positive valence. Decreased positive RT in the near future may reflect the treatment effects by CBT because CBT focuses on “here and now” problems and positive planning for the near future (37). Furthermore, behavioral activation schedules rewarding behaviors into a patient’s routines (38), which may facilitate near future thinking.

Interestingly, improvement of the depressive symptoms and an increase in the ratio of the number of responses for positive valence after the intervention was similar between CBT and TC. These results are in line with a meta-analysis reported by Cuijpers et al. (39) that showed no differences in efficacy among the modalities of psychotherapies for depression. RTs were shortened after the intervention only in the CBT participants in our sample. Summing up these findings, it appears that RT does not reflect an improvement in depressive symptoms.

In our sample, the greater reductions in RTs in the future condition were inversely correlated with the degree of the dysfunctional schema in the CBT group. This finding adds to previous studies (40) that showed that CBT improved dysfunctional schemas and that the improvement of dysfunctional schemas from the use of CBT coincided with the reduction of RT in the future condition. In a previous study investigating information processing associated with DAS responses, healthy individuals showed slowing in RT with agreement with dysfunctional schemas (41). The literature reveals that dysfunctional schemas affect information processing (42), and schema-based information processing is a crucial determinant of the depressed state (43–46).

Interestingly, depressed patients who received CBT were found to show improvements in their need for social approval, as measured by the Social Approval Needs subscale of the DAS. The DAS Need for Social Approval subscale is a measure of interpersonal personality vulnerability (47) and the belief that one’s happiness and self-worth are dependent on the receipt of approval, support, and love from others (48). These findings are consistent with previous studies showing that interpersonal personality vulnerability improved over the course of CBT treatment (47–49).

Strengths of the present study include the use of a rigorous RCT design with a TC as the control group, an FTT to assess changes in future thinking before and after psychotherapy, and a focus on behavioral assessment. While many psychotherapy studies have been based solely on clinical questionnaires (50), the current study was significant because it took a different approach to outcome assessment. In addition, the focus on dysfunctional attitudes suggests that FTT response time captures not only a post-treatment outcome but also the treating process of CBT. However, this study has limitations that should be considered when interpreting the results. First, we had a relatively limited sample size. The lack of significant differences in change pre- and post-intervention between the groups could be due to the effect of the small sample size. Second, participants were recruited from a university teaching hospital, thus limiting generalizability. Third, the FTT may have a potential confounding issue because some aspects of content (e.g., conceptual words or physical-state-related words) were not controlled for. Fourth, most patients in our study received pharmacotherapy for treatment of depression. Therefore, the possibility of medication effects could not be ruled out. Fifth, since the FTT was performed twice, test-retest bias may occur within evaluation periods.

In conclusion, patients with MDD who received a course of CBT experienced reduced RTs in the distant future condition. Therefore, response time to a future-thinking task in depressed patients may contribute as an objective measure for a CBT treatment process. As the present RCT is positioned as a pilot RCT, future research is necessary to further validate the results in a fully powered trial using a larger number of patients and to elucidate the cognitive behavior therapy treatment process that influences future thinking.

## Data availability statement

The original contributions presented in this study are included in this article/Supplementary material, further inquiries can be directed to the corresponding author.

## Ethics statement

The studies involving human participants were reviewed and approved by the Ethical Review Committee of Keio University School of Medicine (reference no. 20150070). The patients/participants provided their written informed consent to participate in this study.

## Author contributions

MA and NK: conceptualization, visualization, project administration, formal analysis, resources, data curation, and writing—review and editing. SU, YT, HT, and TK: investigation, methodology, and writing—review and editing. TA: methodology, formal analysis, and writing—review and editing. MM: project administration, resources, and writing—review and editing. AN: conceptualization, visualization, formal analysis, supervision, project administration, resources, and writing—review and editing. All authors critically reviewed the manuscript for content and approved the final version.

## Footnotes

1
http://www.mhlw.go.jp/bunya/shougaihoken/kokoro/dl/01.pdf
