# Supplementary material for: The effect of cognitive behavioral therapy on future thinking in patients with major depressive disorder: A randomized controlled trial
Source: Front Psychiatry. 2023 Jan 25;14:997154. doi: 10.3389/fpsyt.2023.997154 (PMC9905738; doi:10.3389/fpsyt.2023.997154)
Supplement: Supplementary file 1 [file Table_1.docx]

**Supplementary TABLE A. Main Outcomes at Pre and Post for Patients Receiving Cognitive Behavioral Therapy and Talking Control.**

**1. Response times for positive and negative valence trials in the Future-Thinking Task**

|  | **Cognitive Behavioral Therapy　(n=16)** | | | | | | | | | **Talking Control　(n=15)** | | | | | | | |  | |
| --- | --- | --- | --- | --- | --- | --- | --- | --- | --- | --- | --- | --- | --- | --- | --- | --- | --- | --- | --- |
|  | pre | | post | | Change  (post-pre) | | within groups † | | pre | | | post | | Change  (post-pre) | | within groups † | | between groups ‡ | |
| Measures | Mean | SD | Mean | SD | Mean | SD | t | *p* | Mean | | SD | Mean | SD | Mean | SD | t | *p* | *t* | *p* |
| Response time (s) |  |  |  |  |  |  |  |  |  | |  |  |  |  |  |  |  |  |  |
| For positive valence |  |  |  |  |  |  |  |  |  | |  |  |  |  |  |  |  |  |  |
| Condition Distant Future | 2.8 | 1.5 | 1.8 | 0.8 | -0.9 | 1.3 | 2.9 | **0.012** | 2.5 | | 1.0 | 2.1 | 0.8 | -0.4 | 1.0 | 1.7 | 0.1 | -1.2 | 0.2 |
| Near Future | 2.5 | 0.8 | 1.9 | 0.7 | -0.5 | 0.7 | 2.7 | **0.017** | 2.7 | | 0.9 | 2.1 | 0.7 | -0.6 | 1.2 | 1.8 | 0.09 | 0.2 | 0.9 |
| Near Past | 2.6 | 1.3 | 1.7 | 0.6 | -0.9 | 1.3 | 2.6 | **0.02** | 2.1 | | 0.6 | 2.9 | 3.7 | 0.7 | 3.9 | -0.7 | 0.5 | -1.5 | 0.1 |
| Distant Past | 2.1 | 0.8 | 1.8 | 0.5 | -0.3 | 0.7 | 1.8 | 0.09 | 1.9 | | 0.5 | 1.8 | 0.7 | -0.1 | 0.7 | 0.6 | 0.5 | -0.8 | 0.5 |
| For negative valence |  |  |  |  |  |  |  |  |  | |  |  |  |  |  |  |  |  |  |
| Condition Distant Future | 2.7 | 1.0 | 2.1 | 0.8 | -0.6 | 1.1 | 2.3 | **0.03** | 2.5 | | 0.9 | 2.2 | 1.0 | -0.3 | 1.1 | 1.0 | 0.3 | -0.9 | 0.4 |
| Near Future | 2.4 | 0.7 | 2.0 | 0.6 | -0.4 | 1.0 | 1.4 | 0.2 | 2.2 | | 0.6 | 2.2 | 0.9 | 0.03 | 0.7 | -0.2 | 0.9 | -1.2 | 0.2 |
| Near Past | 2.2 | 0.8 | 1.9 | 0.6 | -0.3 | 0.9 | 1.6 | 0.1 | 2.0 | | 0.5 | 2.0 | 0.6 | 0.03 | 0.6 | -0.2 | 0.8 | -1.4 | 0.2 |
| Distant Past | 2.3 | 0.8 | 2.1 | 0.9 | -0.3 | 0.9 | 1.2 | 0.3 | 2.0 | | 0.7 | 2.1 | 1.2 | 0.06 | 0.9 | -0.3 | 0.8 | -1.0 | 0.3 |

Note: **Bold** items are significant at p<0.05, **†** Paired t-test. **‡** Independent t-test; SD = standard deviation.

**2. Ratio of positive valence trials in the Future-Thinking Task**

|  | **Cognitive Behavioral Therapy　(n=16)** | | | | | | | | **Talking Control　(n=15)** | | | | | | | | |  | | |
| --- | --- | --- | --- | --- | --- | --- | --- | --- | --- | --- | --- | --- | --- | --- | --- | --- | --- | --- | --- | --- |
|  | pre | | post | | Change  (post-pre) | | within groups † | | | pre | | post | | Change  (post-pre) | | within groups † | | | between groups ‡ | |
| Measures | Mean | SD | Mean | SD | Mean | SD | t | *p* | | Mean | SD | Mean | SD | Mean | SD | t | *p* | | t | *p* |
| Ratio of positive valence trials (%) |  |  |  |  |  |  |  |  | |  |  |  |  |  |  |  |  | |  |  |
| Condition Distant Future | 43.4 | 36.1 | 59.4 | 31.3 | 16.0 | 29.9 | -2.1 | **0.04** | | 38.3 | 33.8 | 62.9 | 33.7 | 24.6 | 30.7 | -3.1 | **0.008** | | -0.8 | 0.4 |
| Near Future | 32.4 | 25.1 | 59.8 | 27.4 | 27.3 | 26.8 | -4.1 | **<0.001** | | 23.8 | 15.7 | 55.4 | 34.5 | 31.7 | 29.7 | -4.1 | **0.001** | | -0.4 | 0.7 |
| Near Past | 36.7 | 16.4 | 62.9 | 23.4 | 26.2 | 31.4 | -3.3 | **0.005** | | 30.8 | 24.8 | 59.6 | 28.1 | 28.8 | 30 | -3.7 | **0.002** | | -0.2 | 0.8 |
| Distant Past | 64.1 | 23.9 | 68.4 | 20.1 | 4.3 | 18.6 | -0.9 | 0.4 | | 65 | 17.2 | 65.4 | 14.1 | 0.4 | 13 | -0.1 | 0.8 | | 0.7 | 0.5 |

Note: **Bold** items are significant at p<0.05. **†** Paired t-test. **‡** Independent t-test; SD=standard deviation; % = (number of negative valence responses/16 trials) × 100%.
